# Protocol: Assessing the outcomes and impact of professional doctorate programmes in health and social care on the individual, their profession, their employing organisation and wider society: A comprehensive systematic review

**DOI:** 10.1002/cl2.1446

**Published:** 2024-10-15

**Authors:** Hazel M. Chapman, Robert McSherry, Josette Bettany‐Saltikov, Mridula Mohan, Debbie Spencer

**Affiliations:** ^1^ Faculty of Health, Medicine and Society University of Chester Chester UK; ^2^ School of Health and Life Sciences, Centre for Rehabilitation Teesside University Middlesbrough UK

**Keywords:** doctoral, health and social care, impact, outcomes, postgraduate, professional doctorate, systematic review

## Abstract

This is the protocol for a Campbell systematic review. This review's objectives are to find out (in relation to health and/or social care): (1) What is known about the outcomes and impact of completing (or not completing) a professional doctorate in health and/or social care on the individual professional? (2) What is known about the outcome and impact of completing (or not completing) a professional doctorate in health and/or social care on the employing organisation? (3) What is known about the outcome and impact of completing (or not completing) a professional doctorate in health and/or social care on the profession? (4) What is known about the outcome and impact of completing (or not completing) a professional doctorate in health and/or social care on service users and the wider society? (5) How do we use the findings from this review to inform educators, higher education institutions, professionals, investors in employing organisations and policymakers? (6) What further research will be needed to answer any knowledge gaps or recommendations? (7) Where possible, we will identify and report on any demographic data and discuss their relevance to the impact and outcomes from professional doctorates.

## 1 BACKGROUND

### 1.1 The problem, condition, or issue

As educators, it is important to continue promoting the importance of doctoral education for the development of the individual, their professional practice, policy, education and future research. However, although student outcomes are measured in terms of progression and achievement, minimal work has been completed internationally on the impact of undertaking a doctoral study in terms of personal practice development, organisational benefits or impact on wider policy and society (McSherry et al., 2019).

Where outcomes have been studied, this has been in the form of skills assessment and measurement (Salls et al., 2012). It has been argued that professional doctorates (PDs) have a greater impact on practice than traditional PhDs (Ellis, 2006), but: ‘Although the PD is grounded in professional practice, there is little robust evidence of impact on professional practice and changes in the workplace. More research could usefully be done to explore these impacts’ (Careers Research and Advisory Center, 2016, p. 67).

Theoretically, there are justifications for undertaking different types of doctoral programmes (Taylor & Storey, 2013), yet the major question is aligned to establishing the outcome and impact and how they can be assessed and evaluated (McSherry et al., 2016).

Therefore, the aim of this systematic review is to explore the outcomes and impact of undertaking (whether or not completed) a professional doctorate in health and social care from all perspectives. These perspectives include (but are not limited to):

- the personal and professional for the individual.
- the impact on the person's colleagues, team and organisation the impact on policy, practice and the profession.
- research, knowledge generation and knowledge exchange social, economic and political impact.

For the purpose of this systematic review, our operational definition of a professional doctorate ‘is a doctoral level degree (level 8), combining taught elements and independent research in the student's area of professional practice and expertise’. As with all doctoral degrees, this is the highest level academic qualification that can be achieved.

Examples of programmes included in this definition would be professional doctorates in:

- nursing.
- health and social care.
- social work.
- professional practice in community and social care.
- health.
- occupational therapy.
- dentistry.
- public health.
- pharmacy.

The programme of study may be either full‐time or part‐time, but irrespectively of this, the emphasis is on enabling the student to focus their enquiry on their practice arena. This generally requires the student to work and study contemporaneously. Even students who self‐fund will often need study leave to achieve their doctorate, thus in most cases, the employer will be investing in the professional doctorate student. Most health and social work professionals need to remain professionally active to maintain their professional registration, to stay current and to manage financial demands, for example, to support their families.

Some posts in health and social care education or health practitioner consultant roles stipulate the need to be studying towards or have achieved a relevant doctorate. There is a general consensus surrounding the time taken for completion of professional doctorates:

- full‐time students, 2–5 years.
- part‐time students, 3–8 years.
- 4–6 years for specific professional doctorate students (e.g., doctorates in pharmacy, nursing or social work) to complete their studies.

### 1.2 The intervention

The intervention is undertaking part or all of a professional doctorate in health and/or social care. This will include combined or single programmes across the health and/or social care professions, whether full‐time or part‐time. Undertaking a professional doctorate in health and/or social care is a pre‐existing condition, not an experimental intervention. The reason for including papers where professional doctorate students may not have completed is that understanding why they did not complete, and the impact of undertaking only part of a doctorate, is as important as understanding the impact of achieving a doctorate.

The reasons for undertaking a professional doctorate in health and social care for professionals and/or their employers might include, but are not limited to:

- Self‐development, including self‐esteem, self‐awareness, recognition and rewards by peers.
- Completion of programme and progression in professional roles and activities, for example, evidence of promotion or career progression within or beyond the programme.
- Employer incentives to achieve a doctorate.
- Deeper knowledge and understanding of theory underpinning professional practice to become a more evidence‐informed professional.
- To gain autonomy and seniority within one's profession and discipline.
- Strategic thinking and critical decision‐making skills.
- Efficient and effective leadership and management skills.
- To develop knowledge and education for the profession.
- To engage others in research interests, to influence policy and to have an impact on health, well‐being and social cohesion.
- Capacity‐building within organisation and profession to drive research and development.
- To become a researcher in one's own right, able to identify research needs, design, lead and acquire funding to develop knowledge, practice and policy.
- To improve the quality, process, output and reputation of the employer.
- To enhance the evidence base, standing and reputation of the profession, for example, in terms of publications, presentations, patents, media presence.
- To contribute to national and international advisory and/or regulatory/policy‐making organisations.
- To contribute to the health and social well‐being of society.

It is relevant to know if even commencing such a programme has any effects, and if so, what they are. A number of personal, family, professional and academic variables could potentially have an effect on completion and utilisation of the doctorate. Therefore, it is important to look at completion and progression as well as other outcomes.

Unexpected or undesirable outcomes could include:

- Threats to physical and/or mental wellbeing.
- Loss of self‐esteem or peer‐esteem due to not obtaining a professional doctorate.
- Financial and time costs to self and/or employer.
- Pressure from employers to achieve a doctorate (in a set time period).
- Failure of programme to bring about development or change for individual, employer or the wider society.

### 1.3 How the intervention might work

Through undertaking a doctorate, it is presumed that professionals develop their knowledge, understanding and insights into key concepts underlying their practice and the arena within which they work and that they, therefore, contribute to the body of knowledge for practice. This should enable the individual to progress in terms of the quality of their practice, their contribution to the employing organisation's performance, education and growth, and their impact on the wider society through advising on policy and education. This systematic literature review aims to find evidence to support or refute this presumption. In addition, there is a secondary question as to whether there are any differences in the impact of undertaking a Doctor of Philosophy (PhD) or professional doctorate programme (Boud et al., 2018) in health and social care. Any data where a direct comparison is made will be included in the systematic literature review.

### 1.4 Why it is important to do this review

There are increasing demands by universities for health and/or social care professional academics to undertake a doctorate programme. Senior professionals are sometimes required to achieve doctorates to progress in their careers or wish to further their professional knowledge base and standing.

Together with the rising cost to employers to fund and or support doctoral students (with either finance and study leave or both), it is necessary to demonstrate the value of undertaking a professional doctorate in health and social care in terms of completion, outcomes and impact. The future provision of professional doctorates in health and/or social care is dependent on addressing some fundamental questions. What attributes if anything, they bring to the professionals, their employers and wider society (Cashin, 2018) is still unclear. Therefore, educators need to offer professional doctorate programmes appropriate for future cohorts undertaking this degree (Hawkes & Yerrabati, 2018). In their review of research on professional doctorates, Hawkes and Yerrabati (2018) concluded that there was a paucity of research on professional doctorates other than in education doctorates. There was also a lack of exploration of the wider value that most academics perceive that professional doctorates add. Since professional doctorates are expected to contribute to workplace development (Dos Santos & Lo, 2018), it is important to investigate that claim. Furthermore, given the changing nature of health and social care moving towards integrated service provision, it is incumbent on higher education institutions to be able to evidence both outcomes and impacts of their professional doctorate provision. These need to be able to show the benefits to the individual, the profession, the employers, as well as the wider society. The outcomes and impacts need to provide evidence of both the tangible and non‐tangible performance measures.

## 2 OBJECTIVES

This review's objectives are to find out (in relation to health and/or social care):

- What is known about the outcomes and impact of completing (or not completing) a professional doctorate in health and/or social care on the individual professional?
- What is known about the outcome and impact of completing (or not completing) a professional doctorate in health and/or social care on the employing organisation?
- What is known about the outcome and impact of completing (or not completing) a professional doctorate in health and/or social care on the profession?
- What is known about the outcome and impact of completing (or not completing) a professional doctorate in health and/or social care on service users and the wider society?
- How do we use the findings from this review to inform educators, higher education institutions, professionals, investors in employing organisations and policymakers?
- What further research will be needed to answer any knowledge gaps or recommendations?
- Where possible, we will identify and report on any demographic data and discuss their relevance to the impact and outcomes from professional doctorates.

## 3 METHODS

The overall criteria are shown in Table 1 below and then discussed in the individual sections below.

**Table 1:** Inclusion and exclusion criteria.

	Inclusion criteria	Exclusion criteria
Population	Health and/or social care professionals who have undertaken or completed a professional doctorate in health and/or social care (or a combination of both or one with another subject) that is provided by any Higher Education Institution internationally.	Students who do not belong to any profession within or allied to health and/or social care; Health and/or social care professionals who have not undertaken or completed a professional doctorate in health and/or social care (either not undertaken a doctorate, or undertaken one in an unrelated subject)
Intervention	The intervention is undertaking a professional doctorate in health and/or social care (or a combination of both or one with another subject) that is provided by any Higher Education Institution internationally.	Any other programme of study will be excluded, including PhDs and professional doctorates in other domains (unless they are included in the paper as a comparator to a professional doctorate, which is a possible secondary outcome)
Comparison	Undertaking or completing a professional doctorate in health and/or social care	Not undertaking a professional doctorate in health and/or social care; undertaking a PhD
Outcome	Progression on the programme; completion of the programme; personal and professional costs of and benefits from the programme; employer's costs and gains from the employee undertaking/completing the programme; costs and gains to the profession related to the individual professional undertaking the programme; costs and benefits to society at large.	Any costs or benefits that are not related to undertaking the programme, or that are related to undertaking any programme that is not a professional doctorate in health and/or social care
Study design	Primary research studies will be included. These may be qualitative (including, but not limited to ethnographic, grounded theory, phenomenological, case study or action research studies), quantitative (including, but not limited to randomised controlled trials, clinical controlled trials, quasi‐experimental studies, cohort studies, correlational studies) or mixed methods (pragmatic single‐ or multi‐centre studies using any combination of quantitative and qualitative methods).	Literature reviews of any kind (to prevent double‐accounting); non‐research articles; secondary research
Other criteria	From the year 2000 onwards; English language only	Textbooks; short communications

### 3.1 Criteria for considering studies for this review

#### 3.1.1 Types of studies

Primary research/original studies on professional doctorates in health and social care. All types of empirical research study designs will be included, whether qualitative (including, but not limited to ethnographic, grounded theory, phenomenological, case study or action research studies), quantitative (including, but not limited to randomised controlled trials, clinical controlled trials, quasi‐experimental studies, cohort studies, correlational studies) or mixed methods (pragmatic single‐ or multi‐centre studies using any combination of quantitative and qualitative methods). All studies will be quality appraised using the McGill mixed methods appraisal tool (MMAT) (Hong et al., 2018). Any studies that do not meet the agreed quality criteria will be excluded from the systematic review (see Section 3.4.4 below for further details about the MMAT tool).

#### 3.1.2 Types of participants

The types of participants will be health and/or social care professionals who have undertaken or completed a professional doctorate in health and/or social care (or a combination of both or one with another subject) that is provided by any Higher Education Institution internationally.

#### 3.1.3 Types of interventions

The intervention is undertaking a professional doctorate in health and/or social care (or a combination of both or one with another subject) that is provided by any Higher Education Institution internationally. This can include:

- professional doctorates in: health, public health, social care, social work, nursing, healthcare, allied health or any combination of the above with each other or with related subjects, such as education or business.
- modular‐based or thesis‐based curricula online, blended or in‐person learning.
- single or combined programmes, full‐time or part‐time programmes.
- programmes that are at level 8 (doctoral level) or programmes that are partly at level 7 (Master's level) and partly at level 8 (doctoral level).

#### 3.1.4 Types of outcome measures

##### Primary outcomes

The primary outcomes consist of any benefits accrued to the individual health and social care professional undertaking a relevant professional doctorate, their employing organisation, the profession as a whole and society at large. It is possible to see what some of these might be from the extant literature (see Table 2 below). These are examples, but other outcomes may be found in the course of the systematic literature review. We do not expect to exclude any outcomes that are found within the included papers. We do not have the resources within our funding to translate papers not written in English.

**Table 2:** Primary outcomes (potential, but not limited to).

	Potential benefits (outcomes and impacts)	
Individual	Professional skills and knowledge Employment Status Career Development/Promotions Professional networks Salary Qualifications	Time costs Potential loss of income while studying Potential pressure from employers
Profession	Policy development Capacity building Research innovations Patents	Lack of integration of research knowledge and skills across the professions
Employer/organisation	Improved engagement within the organisation Achievement of organisational goals career longevity Loyalty to organisation Organisational outcomes, for example, greater efficiency, improved services, improved clinical outcomes, greater innovation and enterprise activities	Working hours lost Financial cost Loss of qualified staff to other organisation/roles Dissatisfaction amongst other staff who feel disadvantaged
Research/other	Improved grades Publications and conferences Professional/academic/organisational esteem and credibility	Potential for non‐standardised assessment quality criteria Potentially low rate/quality of publication following completion

##### Secondary outcomes

Where papers include comparative data between professional doctorate and PhD programmes (both for professionals in health and social care) OR between professional doctorates in health and social care and in other subjects (such as education [EdD] or business [DBA]), we will extract that data for inclusion in the review. Thus, there is potential for some comparative analysis of the effects of undertaking different types of doctorate on the variables identified above should the research have been carried out.

##### Duration of follow‐up

There will not be a time limit imposed on follow‐up as impact from undertaking a doctoral degree is expected to have long‐term implications for the individual, their employing organisation, their profession and society.

##### Types of settings

The settings will be a Higher Education Institution that offers professional doctorates in either health or social care or a combined programme, possibly with other subjects, such as education. We are unlikely to exclude programmes from any country unless they differ substantially in content or quality to those programmes found mostly in Australasia, the United Kingdom and in the United States of America and Canada. Learning environments of all types (e.g., online, blended learning, distance learning or in‐person learning) will be included.

### 3.2 Search methods for identification of studies

#### 3.2.1 Electronic searches

**Table:** 

Inclusion criteria	Exclusion criteria
1. Primary research/original studies on professional doctorates in health and/or social care 2. Year of publication: 2000‐2024 3. Language: Publications in English language	1. Studies on professional doctorates and PhDs in other disciplines or professions 2. Systematic reviews 3. Case reports and other communications 4. Textbook chapters 5. Publications before the Year 2000

#### 3.2.2 Search strings

(‘professional doctora*’ OR DProf OR ‘education doctora*’ OR ‘professional studies doctorate’) AND (health OR ‘health and social care’ OR healthcare OR nurs* OR ‘social care’ OR ‘social work*’ OR ‘public health’ OR ‘occupational therap*’ OR dentistry OR pharmacy).

Please see Appendix S1 for pilot search.

Scientific databases:

*APA PsycInfo (EBSCOhost).*

*British Education Index (EBSCOhost).*

*CINAHL Ultimate (EBSCOhost).*

*Education Source (EBSCOhost).*

*ERIC (EBSCOhost).*

*MEDLINE (EBSCOhost).*

*ProQuest Nursing and Allied Health.*

*SCOPUS (Elsevier – scientific abstract and citation database).*

*SocIndex (EBSCOhost).*

*Web of Science (Web of Science Core Collection, ProQuest Dissertations & Theses Citation Index).*

### 3.3 Searching other resources

Ethos (the British Library e‐theses free online service providing access to United Kingdom doctoral theses) (if available).

ProQuest™ Dissertations & Theses Citation Index (Web of Science).

Will include Open Dissertations (EBSCOhost).

Reference lists of included studies – Specific print journals and general searches will not be carried out as it is unlikely to produce new evidence. However, articles included in the review will have their references searched for any papers that meet the inclusion criteria.

We will search *Campbell Systematic Reviews* and Google Scholar to see if any previous reviews have been published on our topic.

Experts in the field will be identified once the included studies have been selected to see if any names appear more often. These authors will then be contacted to see if they know of additional studies that might meet this review's inclusion criteria.

Once screening is complete, Google Scholar will be used to run a citation search on included studies to trace the development of the study as it is cited by subsequent researchers.

### 3.4 Data collection and analysis

#### 3.4.1 Description of methods used in primary research

Primary research/original studies on professional doctorates in health and social care.

All types of empirical research study designs will be included, whether qualitative (including, but not limited to ethnographic, grounded theory, phenomenological, case study or action research studies), quantitative (including, but not limited to randomised controlled trials, clinical controlled trials, quasi‐experimental studies, cohort studies, correlational studies) or mixed methods (pragmatic single‐ or multi‐centre studies using any combination of quantitative and qualitative methods). All studies will be quality appraised using the McGill mixed methods appraisal tool (MMAT) (Hong et al., 2018). Any studies that do not meet the agreed quality criteria will be excluded from the systematic review (see Section 3.4.4 below for further details about the MMAT tool).

#### 3.4.2 Selection of studies

The study selection process will consist of two stages: title/abstract screening and full‐text screening. Four independent reviewers will screen the titles and abstracts of identified articles against the inclusion and exclusion criteria. Disagreements will be resolved through discussion.

During the full‐text screening stage, four independent reviewers will assess the full texts of potentially eligible studies for final inclusion. Any discrepancies at this stage will be resolved by discussion and consensus.

#### 3.4.3 Data extraction and management

A customised data extraction form will be developed to collect relevant information from the included studies. The following data will be extracted:

- Study characteristics: Authors, publication year, journal name, study design, sample size, country. Participant characteristics: demographics (age, gender), professional background.
- Intervention details: type of doctoral studies or professional doctorates, status of completion, status of enrolment.
- Outcome measures: individual level. professional level, organisation/institution level, research level. Key findings: results and conclusions.
- One review author (MR) will collate and enter all data into RevMan Web for review by all authors.

#### 3.4.4 Assessment of risk of bias in included studies

The quality and risk of bias assessment of the included studies will be assessed by four independent reviewers using the McGill mixed methods appraisal tool (MMAT) (Hong et al., 2018). The key criteria include the suitability of data to answer clear research questions; representativeness of participants; appropriateness of data collection method; level of risk of non‐responsiveness and effective integration of study components to answer the research question; use of data to sufficiently substantiate interpretation of results; coherence between data source, collection, analysis and interpretation. Any studies that fail to meet the agreed quality standards will be excluded from the systematic review. The following judgements will be used ‘yes, no, can't tell’. The disagreements will be resolved by discussion and consensus or consultation with an external expert if necessary.

#### 3.4.5 Measures of treatment effect

Measures of treatment effects are not relevant for this study as most of the studies will be qualitative or mixed methods, and there are no standardised outcome measures that can be meta‐analysed. However, any quantitative data will be presented in a tabular or graphic form and discussed accordingly.

#### 3.4.6 Unit of analysis issues

Unit‐of‐analysis refers to the level at which data will be collected and analysed in a study. In this context a possible issue in unit of analysis is at the programme level. It may consider factors such as programme curriculum, structure and resources. Another issue is the organisational level, that is, whether it is a healthcare organisation or social care setting or educational setting.

#### 3.4.7 Criteria for determination of independent findings

To determine independent findings of included studies, the following criteria are considered: data from different study designs will be considered independent.

- If the same data set is used in multiple publications, each publication will be considered independent only if they report unique outcomes or interpretations.
- If multiple studies report findings from the same data set but cover different time periods, we will consider them as independent findings.
- If the same research team publishes multiple studies, it will be thoroughly verified and, if they provide additional unique findings or additions insights, these will be considered as independent findings.

#### 3.4.8 Dealing with missing data

First of all, during the data extraction process, the extent and presence of missing data in the included studies will be properly documented. The reasons for missing data such as participant attrition, incomplete reporting, non‐response or other reason will be documented. It may be provided by the original study authors themselves in the paper, otherwise, where possible, authors of the included studies will be contacted to obtain any missing data.

#### 3.4.9 Assessment of heterogeneity

Heterogeneity will be assessed by four authors, and meta‐analysis will only be conducted where all of them agree that study participants, interventions and outcomes are sufficiently similar. Statistical heterogeneity will be assessed using the *I* ^2^ statistic, should sufficient quantitative papers, using similar types of data, be found. In terms of qualitative heterogeneity, themes will be discussed, but any anomalies or unexpected findings will be highlighted, as would be expected in any qualitative analysis.

#### 3.4.10 Assessment of reporting biases

Where possible, the authors of the included studies will be contacted to enquire about any unpublished data or ongoing studies that may be relevant to the review. Authors may provide additional information or unpublished results that can help assess reporting bias.

The articles will also be evaluated for selective outcome reporting. This will be done by comparing the reported outcomes in the methods section with those reported in the results section. Discrepancies may indicate selective reporting bias, where only statistically significant or favourable outcomes are reported.

#### 3.4.11 Data synthesis

A narrative synthesis approach will be used to summarise and integrate the findings from the included studies. The extracted qualitative data will be analysed thematically, identifying common themes, patterns and variations in the impact of doctoral studies or professional doctorates in health and social care. If feasible and appropriate, a meta‐analysis will be conducted to pool quantitative data using appropriate statistical techniques. For any outcomes for which the included studies are not sufficiently homogeneous, or for which insufficient data are found for meta‐analysis, a narrative synthesis will be presented.

#### 3.4.12 Subgroup analysis and investigation of heterogeneity

The following sub‐groups will be investigated, if possible:

- PhD versus Professional doctorates.
- Professional doctorates in other fields for professionals in health and social care (e.g., EdD or DBA) versus Professional doctorates in health and/or social care.

#### 3.4.13 Sensitivity analysis

Sensitivity analyses will be conducted to assess the effect of risk of bias in the included studies, comparing studies rated at high or low risk of bias in the MMAT tool for each assessed item.

#### 3.4.14 Treatment of qualitative research

All qualitative primary research studies will be assessed using the MMAT tool (Hong et al., 2018) and synthesised using thematic analysis according to Braun and Clarke's method (Braun & Clarke, 2022). This method has been described in Byrne's chapter on the application of this approach to data analysis (Byrne, 2022) and is used in a systematic review and thematic analysis by McCluskey et al. (2022).

#### 3.4.15 Summary of findings and assessment of the certainty of the evidence

The thematic analysis and narrative synthesis of the findings will be summarised in a logical and meaningful way. Following data extraction and quality assessment, we will carry out an initial coding building to the development of overall themes related to our review questions, using Braun and Clarke's (2022) method. We will focus on the strength of the evidence to achieve the following:

- What is known about the outcomes and impact of completing (or not completing) a professional doctorate in health and/or social care on the individual professional?
- What is known about the outcome and impact of completing (or not completing) a professional doctorate in health and/or social care on the employing organisation?
- What is known about the outcome and impact of completing (or not completing) a professional doctorate in health and/or social care on the profession?
- What is known about the outcome and impact of completing (or not completing) a professional doctorate in health and/or social care on service users and the wider society?
- How do we use the findings from this review to inform educators, higher education institutions, professionals, investors in employing organisations and policy makers?
- What further research will be needed to answer any knowledge gaps or recommendations?

## CONTRIBUTIONS OF AUTHORS

Dr. Hazel M. Chapman – project lead, expertise in content of doctoral education (professional doctorate in health and social care programme lead) systematic review methods and content; experience of information retrieval; some statistical analysis skills.

Professor Robert McSherry – expert in both content and systematic review methods – author on both aspects of study – advising project lead.

Associate Professor Josette Bettany‐Saltikov – expert in both content and systematic review methods – author on both aspects of study – advising project lead.

Debbie Spencer, Subject Librarian – expert in information retrieval and management.

Dr. Mridula Mohan – helped with initial development of literature search strategy; expert in systematic review methods (trained at Joanna Briggs Institute); expert in statistical analysis.

- Content: Dr. Hazel M Chapman; Professor Robert McSherry; Dr. Josette Bettany‐Saltikov.
- Systematic review methods: Professor Robert McSherry; Dr. Josette Bettany‐Saltikov; Dr. Hazel M. Chapman.
- Statistical analysis: Dr. Hazel M. Chapman.
- Information retrieval: Debbie Spencer; Dr. Hazel M. Chapman.

## DECLARATIONS OF INTEREST

No, however, we are using this project as information to support two qualitative studies on the impact of undertaking a professional doctorate in health and social care, and it is possible that these may be submitted for publication before this literature review being accepted for publication.

## PRELIMINARY TIMEFRAME

We expect to submit the review within 1 year of acceptance of the protocol.

## PLANS FOR UPDATING THIS REVIEW

The team will be responsible for updating this every 2 years.

## DATA AND ANALYTIC CODE

No new data were created or analysed in this study. Data sharing is not applicable to this article.

## SOURCES OF SUPPORT

### Internal sources

An internal grant from the University of Chester was awarded to cover some of the researchers' time and the cost of COVIDENCE systematic review software for 1 year.

### External sources

N/A.

## Supporting information

Supporting information.
