# Supplementary material for: Protocol: Assessing the outcomes and impact of professional doctorate programmes in health and social care on the individual, their profession, their employing organisation and wider society: A comprehensive systematic review
Source: Campbell Syst Rev. 2024 Oct 15;20(4):e1446. doi: 10.1002/cl2.1446 (PMC11473971; doi:10.1002/cl2.1446)
Supplement: Supplementary file 1 — Supporting information. [file CL2-20-e1446-s001.docx]

# Appendices

##### Appendices should be submitted as [supplementary material](https://authorservices.wiley.com/author-resources/Journal-Authors/Prepare/manuscript-preparation-guidelines.html/supporting-information.html).

## Appendix 1 Search Strategy:

MEDLINE (EBSCOHost) search strategy – pilot search August 2024

| # | Searches | Results |
| --- | --- | --- |
| 1 | "professional doctora*" | 114 |
| 2 | DProf | 3 |
| 3 | "education doctoral" | 27 |
| 4 | “professional studies doctorate” | 1,384 |
| 5 | 1 or 2 or 3 or 4 | 304 |
| 6 | Health | 6,782,543 |
| 7 | “health and social care” | 17,243 |
| 8 | healthcare | 718,320 |
| 9 | Nurs* | 1,205,723 |
| 10 | MH "Nursing+" | 266,559 |
| 11 | MH "Nurses+" | 101,076 |
| 12 | “social care” | 67,289 |
| 13 | “social work*” | 83,628 |
| 14 | MH "Social Work+" | 18,972 |
| 15 | “public health” | 1,484,574 |
| 16 | MH "Public Health+" | 9,614,427 |
| 17 | “occupational therap* | 51,524 |
| 18 | MH "Occupational Therapy+" | 15,438 |
| 19 | dentistry | 513,665 |
| 20 | MH "Dentistry+" | 443,362 |
| 21 | pharmacy | 778,156 |
| 22 | MH "Pharmacy+" | 10,734 |
| 23 | 6 or 7 or 8 or 9 or 10 or 11 or 12 or 13 or 14 or 15 or 16 or 17 or 18 or 19 or 20 or 21 or 22 | 14,729,802 |
| 26 | 5 AND 23 | 186 |
